# Serum heat shock protein 70 in preeclampsia and normal pregnancy: A systematic review and meta-analysis

**Published:** 2018-01

**Authors:** Nafiseh Saghafi, Leila Pourali, Vahid Ghavami Ghanbarabadi, Fatemeh Mirzamarjani, Masoumeh Mirteimouri

**Affiliations:** 1 *Department of Obstetrics and Gynecology, Faculty of Medicine, Mashhad University of Medical Sciences, Mashhad, Iran.*; 2 *Department of Epidemiology and Biostatistics, School of Public Health, Tehran University of Medical Sciences, Tehran, Iran.*

**Keywords:** Heat shock protein 70 (HSP70), Preeclampsia, Pregnancy, Meta-analysis

## Abstract

**Background:**

Preeclampsia, a severe complication of human pregnancy is one of the main causes of maternal, fetal, and neonatal morbidity and mortality with unclear pathogenesis. Heat shock protein 70 (HSP70) is one of the factors that can mediate cytoprotective, antiapoptotic, and immune regulatory effects.

**Objective:**

This meta-analysis was performed with aim to evaluate HSP70 in preeclampsia and normal pregnancy.

**Materials and Methods:**

The original publications reporting the serum HSP70 levels in preeclampsia and normal pregnancies published before November 2015 were identified by searching PubMed Central, Scopus, and ISI Web of Knowledge databases by two researchers, separately. The keywords were” preeclampsia” and “HSP70” or “Heat shock protein 70” Statistical analyses were performed using STATA software (version 11).

**Results:**

Out of 127 studies, seven eligible case-control studies were identified which consists of 350 preeclampsia and 429 normal pregnancies. Our pooled analysis of data from 7 studies which met the inclusion criteria, provides evidence that there is a significant association between HSP70 and preeclampsia. Cochran's test results showed the heterogeneity of the studies (p<0.001) and the I2 index was 91%. The standardized mean differences (SMD) based on a random effect model with trim and fill method was 0.92 (95% CI: 0.33-1.51); also there was a significant association between HSP70 and preeclampsia (Z=3.07, p=0.002).

**Conclusion:**

The results showed that serum HSP70 concentration was significantly higher in preeclamptic patients than the control group. Therefore HSP70 may be identified as a diagnostic factor.

## Introduction

Preeclampsia, a multisystemic disorder, is one of the most critical complications of human pregnancy. It is one of the main causes of maternal, fetal, and neonatal mortality, especially in low-income and middle-income countries, affecting 3-5% of pregnancies (1, 2). 

Despite the existence of many scientific studies worldwide, the etiology and pathogenesis of preeclampsia is unclear and remains a challenge. The disease is characterized by hypertension (BP ≥140/90 mmHg) and proteinuria (>300 mg per day) developing after the 20^th^ wk of gestation (3). It may be a collection of syndromes with different precipitating factors and outcomes. Studies have suggested that previous history of preeclampsia, antiphospholipid syndrome, family history of hypertension, nulliparity, chronic kidney disease, insulin-dependent diabetes, multiple pregnancies, and preexisting hypertension are well known as risk factors for preeclampsia (4). Some studies have indicated that enhanced maternal systemic inflammatory responses to pregnancy with the activation of the immune system have an important role in the pathogenesis of this syndrome (5). Also, several studies have identified gene variants involved in thrombophilia, inflammation, oxidative stress, and the renin-angiotensin system in relation to preeclampsia (6). Therefore, the factors involved in these pathways may be identified as the diagnostic factors. 

Heat shock proteins (HSP), are highly conserved molecules and major molecular chaperones that increase in response to a variety of stress stimuli and restore protein homeostasis (7). HSP70 is one of the major HSPs that is essential for a cell's machinery and is involved in numerous processes including protein folding. The intracellular inducible HSP70 can mediate cytoprotective, antiapoptotic, and immune regulatory effects (8). Several studies have indicated that HSP70 has a role in the pathogenesis of hypertension and associated diseases (9). Also, high levels of HSP70 have been shown in serum, plasma and placental tissue of preeclamptic patients (10). Placental ischemia, oxidative stress, and maternal systemic inflammatory response, which are major elements in the pathogenesis of preeclampsia, have been shown to induce the expression of HSP70. A large number of case-control studies have been conducted to explore the association between serum levels of HSP70 and preeclampsia (11-19). 

However, the results are inconsistent: most of such studies have only modest sample sizes, and this limits their significance. In some studies, HSP70 in preeclampsia was significantly higher than normal pregnancy; even some studies showed that it is much higher in pregnancies complicated with early onset than late-onset preeclampsia (14, 16, 20, 21). 

On the other hand, some studies didn’t find significant higher serum level of HSP70 in preeclampsia compare to normal pregnancy (18, 22). By performing a meta-analysis and providing a prevailing method for the quantitative summary of different results, the data may be assessed and the sample size can be increased to a reasonable level. 

In the present study, a meta-analysis was designed to quantitatively assess the association between the maternal serum levels of HSP70 and preeclampsia.

## Materials and methods

**Identiﬁcation of studies for meta-analysis**

The original publications reporting the serum HSP70 levels in preeclampsia and normal pregnancies published before November 2015 were identified by searching PubMed Central, Scopus, and ISI Web of Knowledge databases by two researchers, separately. Two researchers independently screened titles, abstracts, and keywords by searching “preeclampsia” and “Heat Shock Protein 70” or “HSP70” as keywords. The references listed in the retrieved articles were also searched simultaneously to find additional eligible studies. 

The articles selected for the analysis used case-control design and were in the English language. The selected articles had a specified sample size, heat shock protein concentration, and distribution indices. Exclusion criteria were duplicated articles, narrative reviews, case reports, and letters to editors. The articles which have not written in English were also excluded. Electronic searching was supplemented by hand searching of the reference list.

**Data extraction**

Meta-analysis was conducted according to PRISMA checklists. The features of the selected studies were independently extracted by two researchers. For each study, the publication date, first author, number of cases and controls, and HSP70 concentration were extracted, and the results were reviewed by a third investigator. 

**Statistical analysis**

Because some studies had used the median in their results and the others had reported the results through the mean, the first step was to convert the different indices to a unique format and then calculate the standardized mean differences (SMD) and its confidence interval using Hedges’ adjusted g method to determine the difference between HSP70 concentration in pregnancy groups (normal and preeclampsia). 

A graphic representation of the results and weight of the studies was demonstrated in a forest plot. The heterogeneity of the information in different articles was evaluated using the Cochran heterogeneity test and I2 index. The pooled index was calculated using the Der Simonion and Laird method (23). In order to indicate the source of heterogeneity, meta-regression analysis was done. Because of the few available studies and the lack of some parameters in some of the articles, the analysis was individually done on each variable. To assess the publication bias, a funnel plot was chosen, and the Trim and filled method (24) were used to test and adjust for possible publication bias. All analysis was performed using STATA software version 11. 

## Results

In total, 129 records were identified from databases. After excluding duplicated and unrelated papers, 7 case-control studies concerning the comparison of HSP70 between preeclampsia and normal pregnancy were identified (Figure 1). The identified studies included 350 preeclamptic cases and 429 controls. The sample size in the case-control studies was considerably varied (ranging from 7 to 67 individuals in each case group) (Table I).

Among the 7 final studies, 3 showed the results related to HSP70 serum concentration in the groups of normal pregnancy and preeclampsia types in mean and standard deviation, and 3 illustrated the data in interquartile range and median. The remaining study displayed the results in range and median values. To complete the meta-analysis, the investigated indices needed to be homogenized; therefore, the following formula was used to homogenize the data by converting the median index to the mean (20).

$\bar{x}\approx \frac{a+2m+b}{4}$

$S^{2}\approx \frac{1}{12}\left(\frac{{\left(a-2m+b\right)}^{2}}{4}+{\left(b-a\right)}^{2}\right)$

$S_{D}=\frac{\mathrm{IQR}}{1.35}$

In this formula, a, b, m and IQR are minimum, maximum, median, and interquartile range respectively.

**Figure 2:**
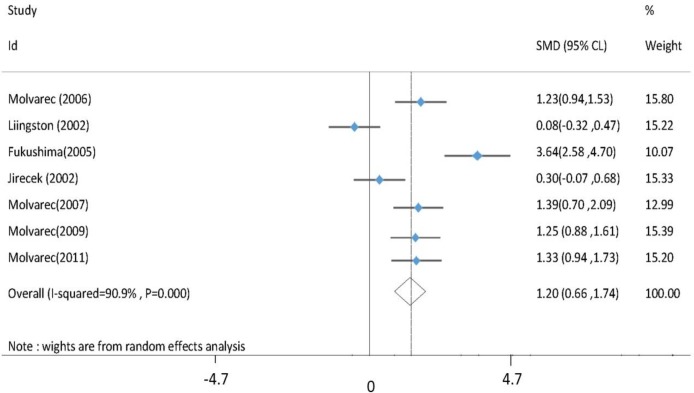
Forrest plot of SMD

Cochran's test results showed the heterogeneity of the studies (p<0.001), and the I2 index was 91%; therefore, a model using the Der Simonion and Laird random effect was adopted. These results showed that the serum HSP70 concentration was significantly higher in preeclamptic patients than in the control group (Z=4.35, p<0.001). The pooled index of standardized mean differences (SMD) of HSP70 serum concentration in the two groups with a confidence interval of 59% was 1.20 (0.66-1.74). The forest plot is shown in figure 2. 

**Figure 3:**
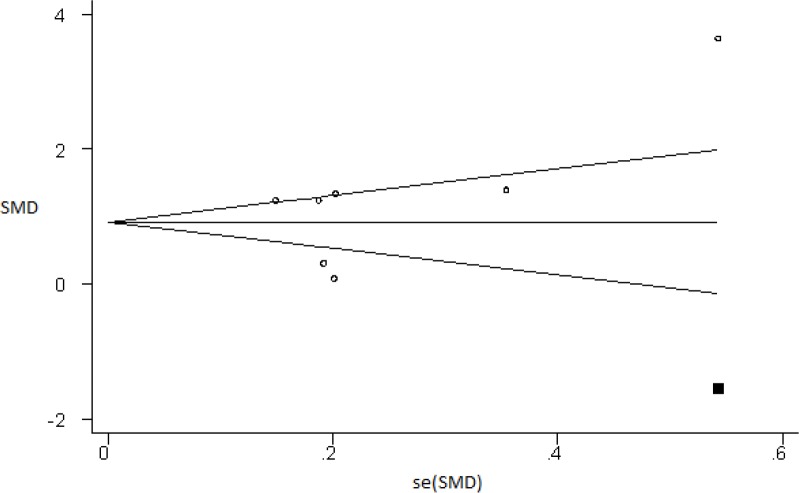
Funnel plot with one imputed filled value (shown by square).

The results of meta-regression showed that gestational age at the time of sampling and maternal age were the sources of heterogeneity with an error level of 0.05. We used trim and fill, a funnel-plot-based method, to evaluate for possible publication bias in our analysis (Figure 3). To evaluate the publication bias, Egger test was used which showed no publication bias (p=0.32). The adjusted summary index with a confidence interval of 95% in the trim and fill method was 0.92 (0.33-1.51); furthermore, the serum HSP70 concentration was significantly higher in preeclamptic patients than in the control group (Z=3.07, p=0.002). It should be noted that because of the low number of studies, the results of the meta-regression and the funnel plot should be interpreted warily.

**Table I:** Studies used in the meta-analysis and extracted data

**Studies**	**Number**		**Age**		**HSP70**		**BMI**		**Gestational age at blood draw**		**Gestational age at delivery**		**Systolic blood pressure at blood draw**		**Diastolic blood pressure at blood draw**		**Fetal birth weight**	
	**Cases**	**Controls**	**Cases**	**Controls**	**Cases**	**Controls**	**Cases**	**Controls**	**Cases**	**Controls**	**Cases**	**Controls**	**Cases**	**Controls**	**Cases**	**Controls**	**Cases**	**Controls**
**Jirecek S (11)**	55	55	-	-	2.82 ± 8.33	1.01 ± 1.38	**-**	**-**	**-**	**-**	**-**	**-**	**-**	**-**	**-**	**-**	**-**	**-**
**Jeffrey C. Livingston (18)** *	48	51	-	-	35.4 ± 96.7	30.1 ± 11.5	-	-	34.3 ± 5.6	35.2 ± 6.0	-	-	174.7 ± 17.2	174.7 ± 17.2	110.0 ± 12.1	79.7 ± 13.8	-	-
**Akimune Fukushima (19)** *	7	46	34.0 ± 1.4	28.7 ± 0.7	24.4 ± 3.6	6.1 ± 0.6	-	-	33.4 ± 2.0	24.7 ± 1.5	33.9 ± 2.0	39.5 ± 0.1	-	-	-	-	-	-
**Attila Molvarec (16)** **	93	127	28 (25–32)	28 (25–31)	0.55 (0.42–0.80)	0.31 (0.27–0.39)	29.4 (26.3–32.0(	26.0 (23.7–28.0)	37 (35–39)	35 (31–37)	38 (35–39)	40 (39–40)	170 (160–180)	110 (105–120)	104 (100–115)	70 (60–80)	2900 (1980–3450)	3300 (3100–3800(
**Attila Molvarec (17)** **	20	20	27 (23–32.5)	28.5 (26–32)	0.54 (0.47–0.79)	0.31 (0.27–0.39)	21.5 (19.7–25.5)	22.0 (19.7–24.2)	33 (30–34)	33 (30–35)	33.5 (32.5–35)	39(38–40)	180 (170–190(	120 (110–120)	120 (110–125)	70 (70–80)	1665 (1160–2075)	3475 (3100–3650)
**Attila Molvarec (15)** **	67	70	29 (19–42)	30 (17–44)	0.58 (0.15–3.47)	0.28 (0.03–0.59)	30.0 (20.6–38.3)	25.9 (19.0–42.0	38 (30–41)	35 (20–40)	39 (35–41)	38 (33–41)	160 (135–220)	110 (80–138)	100 (90–131)	70 (55–86)	3200 (1400–4200)	3500 (2650–4400)
**Attila Molvarec (14)** **	60	60	29 (26–32)	30 (28–32)	0.58 (0.39–0.81)	0.28 (0.21–0.31)	29.9 (26.9–33.3)	25.8 (24.3–27.9)	37 (36–39)	36 (36–37)	38 (37–39)	39 (38–40)	162 (155–180)	110 (107–120)	100 (97–110)	70 (60–80)	3125 (2450–3475(	3450 (3150–3700)

HSP70: Heat Shock Protein 70

BMI: Body Mass Index

* Values were reported in mean±SD

** Values were reported in median (Q1-Q3)

*** Values were reported in median (min-max)

**8 Records were remained 1 article was excluded because it has repetitive data 53 Records were excluded for improper title/abstract60 Records were remained Figure 1.**
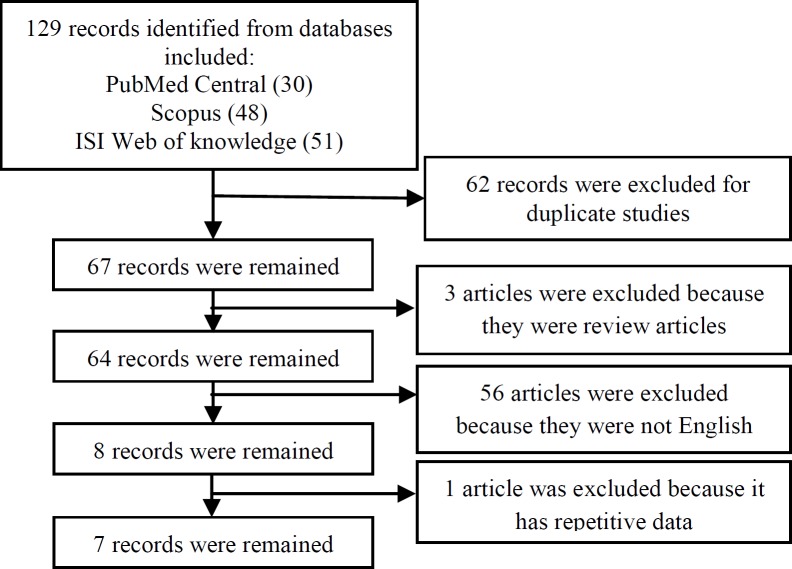
The selection process of studies

## Discussion

Despite the many advances in early detection and treatment of preeclampsia, its prognosis remains a very important problem in perinatal medicine. Because some evidence exists investigating the role of oxidative stress in preeclampsia, researchers have studied many factors such as heat shock proteins to resolve this problem. However, the results of these studies are heterogenic and inconsistent. Previously, Molvarec *et al* evaluated the serum levels of HSP70 in 142 hypertensive pregnant women and 123 normal pregnancies and found higher serum levels of HSP70 in hypertensive women compared with healthy controls (16). 

Peracoli *and colleagues* also conducted a similar study which demonstrated the higher serum levels of HSP70 in patients complicated by early onset preeclampsia (20). Again, Molvarec *et al* in 2011 performed a case-control study which evaluated HSP70, circulating cytokines, chemokines, adhesion molecules and angiogenic factors in pregnant women with preeclampsia and showed increased serum HSP70 concentration in preeclamptic women were associated with proinflammatory changes in circulating cytokine profile (14). In contrast, Akbarzadeh-Jahromi and coworkers found no significant difference between circulating levels of HSP70 in women with preeclampsia and healthy pregnant women (22). 

Hozo *et al* (25) and Livingstone and colleagues (18) also didn’t find a significant difference in serum levels of HSP70 in hypertensive and normal pregnant women. On the other hand, because the sample sizes of these studies are small, may be the results are underpowered statistically. It seems that in order to evaluate the relation of these factors with preeclampsia precisely, one needs to perform well-designed studies and collect pooled data with a large sample size. In this study, we designed a meta-analysis study to evaluate the association of serum HSP70 concentration and preeclampsia. 

Our pooled analysis of data from 7 studies which met the inclusion criteria included 350 preeclamptic cases and 429 controls. Pooled data provided evidence that there is an association between HSP70 and preeclampsia. These results showed that the serum HSP70 concentration was significantly higher in preeclamptic patients than in the control group. This result is in line with studies that have shown that expressions of HSP70 increase in a hypertensive person. Also, previous studies indicated that pre-hypertensive conditions induce HSP70 expression in tubular cells, blood vessels, and microglia of the central nervous system (9).

According to existing evidence that shows a high concentration of HSP70 is associated with the cytokine profile, high levels of HSP70 may occur in response to systemic inflammation, oxidative stress, and hepatocellular injury in preeclampsia (15). In fact, it seems that preeclampsia is a part of the severe inflammatory response of a pregnant mother, so it can be expected that HSP70 expression is induced in response to stress and thus shows increased concentration in preeclamptic patients compared to healthy subjects. Therefore, using HSP70 to identify patients with preeclampsia is not only a useful marker, but it also plays a role in the pathogenesis of the disease. 

Because our data is heterogenic, we propose to perform a well-designed study with a larger sample size to evaluate this association precisely. However, because the confounding factors such as body mass index and age can affect the results, we suggested that these factors be removed or matched in two case and control groups. On the other hand, genetic variation in the HSP70 gene can affect the level of its expression. In terms of the different genetic basis of population, we suggested that analysis of these variants in relation to the serum level of HSP70 be investigated. Although we used meta regression to indicate the source of heterogeneity because of the small number of studies, there is a great danger of over fitting, and many characteristics of the studies may be identified as potential causes of heterogeneity. 

On the basis of our research, this paper is the first meta-analysis in this subject, so it is the most important strength of current research. One of the weaknesses of this study is an evaluation of only English articles; as a result, some studies were excluded; so, much data was missed. 

Another limitation is that because of the low number of studies (less than 10 studies), the use of technical approaches such as meta-regression and funnel plot was not recommended; however, these methods were used with emphasizing to this limitation. The other limitation is related to the meta-analysis itself which cannot show the causality effect. Therefore, it is recommended that future studies be conducted based on the standard protocol. 

## Conclusion

In conclusion, our study indicated that serum levels of HSP70 are significantly higher in preeclampsia than normal pregnancies. 

## Note

***This article extracted from M.D. Thesis. (Fatemeh Mirzamarjani)***
